# Impact of climate change on pediatric health outcomes

**DOI:** 10.1080/16549716.2026.2648401

**Published:** 2026-04-15

**Authors:** Mohammed Abbas, Dhafer Obead Alqahtani

**Affiliations:** a Department of Pediatrics, College of Medicine and Health Sciences, Arabian Gulf University, Manama, Bahrain; b Pediatric Cardiology and Advance Cardiac Imaging Consultant, Head of Advanced Cardiac (CT/MRI) Unit Prince Sultan Cardiac Center Riyadh, Bahrain

**Keywords:** Climate change, pediatric health, air pollution, infectious diseases, systematic review

## Abstract

Climate change has become one of the most critical health issues globally in the twenty-first century with children bearing the disproportionate burden of the burden since they are more vulnerable than adults because of their physiological, behavioral, and developmental capacities. It is a systematic review that rates the evidence of the relationship between climatic exposures such as heat, air-pollution, and extreme weather events and pediatric health outcomes. The number of peer-reviewed studies involved was 23 published in 2000–2025, which represented different geographic areas and study designs and assessed acute and chronic health outcomes. The Newcastle-Ottawa Scale and the ROBINS-I tool were used to evaluate the methodological quality, and the majority of the studies had low to moderate risks of bias. The narrative synthesis shows that there are always links between air pollutants especially PM2.5, NO2 and O3 and respiratory morbidity, prevalence of asthma and hospitalization of children. Amplified temperatures as well as heat waves were associated with increased cases of heat illness, dehydration, and febrile state in infants and young children. There were elevated cases of diarrheal and vector-related infections, especially in low-resource settings, which were linked to extreme weather events especially floods. Although the overall results were similar, significant differences in the regions and methods were found, and low-income countries show little evidence. In addition, exposures as analyzed in most studies were usually considered individually, which may have underestimated the cumulative or compound climate risks.

## PAPER CONTEXT

- **Main findings:** This review shows that rising temperatures, air pollutants, and extreme weather events are consistently linked with higher rates of respiratory illness, heat-related illness, and infectious diseases among children.
- **Added knowledge:** The synthesis brings together two decades of evidence showing that children across multiple continents experience similar health vulnerabilities when exposed to climate-related environmental conditions.
- **Global health impact for policy and action:** The findings highlight an urgent need for stronger climate-responsive child health policies and early warning systems to protect children from worsening environmental threats worldwide.

## Background

Global warming has become one of the most important environmental and human health problems of the twenty-first century [1]. Due to anthropogenic greenhouse gas emissions, which have disrupted ecosystems, altered weather patterns, and increased the transmission of infectious diseases, the global average surface temperature has increased at a rate unheard of in modern history [2]. The Intergovernmental Panel on Climate Change (IPCC) estimates that current emission trends will raise global temperatures by 1.5–2°C by the middle of the century, with dire human health effects [3]. Children are the most vulnerable group among affected populations, as they are particularly sensitive to environmental stressors due to their physiological, behavioral, and developmental characteristics. The increased frequency and severity of air pollution incidents, floods, droughts, and wildfires are intertwined hazards that endanger children’s health worldwide [4].

Children’s susceptibility to climate change is due to a constellation of biological and social determinants. Physiologically, they have elevated metabolic and respiratory rates relative to body mass, incomplete thermoregulatory systems, and underdeveloped immune and detoxification mechanisms, which predispose them to environmental toxins and thermal effects [5]. Children are also more at risk due to their behavioral patterns, which are more outdoor and reliant on their caretakers to ensure their safety and well-being, including healthcare services. Such vulnerabilities are further increased by socioeconomic factors like poverty, poor housing, urban heat island effects, and poor access to healthcare, which particularly burden children in low-resource environments. This biological immaturity and social disadvantage place the children at the centre of the climate-related health risks [6].

Empirical evidence shows that climate change negatively impacts children’s health in various direct and indirect ways. Health-related diseases, respiratory ailments, communicable illnesses, malnutrition, and psychological consequences constitute the short-term effects [7]. The hot ambient temperatures have been linked with a rise in pediatric emergency department visits and hospitalized cases of dehydration, heat stroke, and exacerbation of chronic respiratory diseases like asthma. Increased ambient temperature also increases the formation of ground-level ozone and concentration of particulate matter, which are the strong predictors of asthma exacerbations, a major cause of child morbidity on the international scene [8]. A rise in temperature (a 1°C increase above local averages) has been associated with increased hospitalizations for asthma in children, supporting the respiratory system’s sensitivity to thermal and pollution stressors in children [9].

Alterations in the ecology and epidemiology of infectious diseases are also determined by changes in temperature and precipitation patterns. The increase in climatic temperatures is exposing children to the risks of contracting vector-borne diseases such as dengue, malaria, and Zika virus infection, due to the expansion of vector habitats for Aedes and Anopheles mosquitoes [10]. At the same time, flooding and intense precipitation increase exposure to diarrheal pathogens through contaminated water sources, and droughts and agricultural upheavals disrupt food security, resulting in undernutrition, stunting, and micronutrient deficiencies. Also, heat during prenatal and perinatal periods has been linked to negative birth outcomes such as preterm birth, low birth weight, and stillbirths, and this indicates that the manifestation of climate change starts in the prenatal stage and extends to later stages of development [11].

Quantitative knowledge of the climate-related effects on pediatric health remains incomplete despite the increasing amount of evidence. The various studies differ in the exposure measures, definitions of outcomes, geographical regions of interest, and the rigor of the methodologies, making it hard to compare and synthesize findings across settings [12]. Most of the available studies are skewed towards high-income nations, and there is a big gap in information in the low- and middle-income areas where children are compounded because they experience poverty, malnutrition, and a diminished adaptive capacity. In addition, although some systematic reviews have isolated exposure-outcome pairs, e.g. the effect of heat on hospital admissions or the effects of air pollution on asthma, the majority are narrative rather than quantitative. This deficiency in extensive meta-analytic synthesis constrains the capacity of policy-makers and healthcare planners to give priority to interventions or allocate resources [13].

More insight into the impacts of climate change on child health is critical to achieving sustainable development and climate resilience objectives. The future generation of children will be even more affected by the health impacts of global warming than previous generations [14]. The World Health Organization (WHO) estimates that children below the age of five already contribute more than 80% of the disease burden worldwide attributable to the environment, a figure likely to increase with climate change [15]. This is not evenly spread, as the children of low-income, marginalized, and urban poor citizens are at the most risk because they are already unevenly disposed to health infrastructure, exposure to the environment, and adaptability. To remedy these inequities, it will be necessary to evaluate evidence-based transformations of different climate exposures, including heat, air pollution, and extreme weather, into pediatric health outcomes in different settings.

The evidence base can therefore only be evaluated systematically and quantitatively in developing adaptive public health strategies. The quantification of the strength and inconsistency of relationships between exposure to climate-related risks and childhood health outcomes can inform policymakers and health professionals in implementing context-sensitive programs (e.g. early warning systems for heat stress, climate-resistant health facilities, and community-based programs to adapt to the climate). In addition, the exposure-outcome relationships with limited prior research would be discovered, which can then inform future research investments and capacity-building [16].

It is in light of such considerations that this research intends to conduct a systematic review and quantitative synthesis of the available evidence worldwide on the relationship between climate change-related exposures and health outcomes in pediatrics. Its specific objectives include the definition and classification of the scope of climate-related exposures under analysis regarding children’s health, the estimation of key outcomes of heat-related illness, respiratory illness, and infectious illness, the degree of heterogeneity across geographic regions and study designs, and the situational evaluation of the quality of the evidence base. The results aim to inform evidence-based pediatric, environmental, and policy responses in order to safeguard children in a changing climate.

### Research design

The research design adopted a systematic review to synthesize available evidence on the relationship between climate change-related exposures and pediatric health outcomes worldwide. To make the research transparent, reproducible, and methodologically rigorous, it was conducted in accordance with the guidelines of the Preferred Reporting Items for Systematic Reviews and Meta-Analyses (PRISMA 2020). The general idea behind this strategy was to determine, evaluate, and quantitatively combine epidemiological evidence on the effects of heat exposure, air pollution, and extreme weather events on child health across various geographic and demographic settings.

### Data collection and search strategy

The data collection method was thorough and systematic, as several academic and grey literature sources were used. A systematic search strategy was used across major databases (PubMed, Scopus, Web of Science, and ScienceDirect) to identify publications published since January 2000 to July 2025. The search was conducted using controlled vocabulary terms (MeSH) and free-text terms related to climate change, global warming, air pollution, extreme temperatures, flooding, drought, pediatric, and child health. The search results were narrowed using Boolean operators to include all potentially relevant literature.

The reference lists of all retrieved studies and of other pertinent systematic reviews were manually filtered to identify additional eligible publications and complete the list. WHO and UNICEF reports, government technical documents, and academic theses were also reviewed to minimize publication bias. EndNote X9 software was used to filter all search results and eliminate duplicates. Two reviewers screened the titles and abstracts of the other studies against predetermined eligibility criteria. Reviews were then conducted in full to ensure inclusion; any discrepancies among the reviewers were resolved in a discussion with a third reviewer, who was consulted as needed.

### Inclusion and exclusion criteria

The inclusion and exclusion procedures were based on clear criteria aimed at ensuring that only methodologically sound and relevant studies were selected for the research objectives. Peer-reviewed observational studies, i.e. cohort, case-control, and time-series studies, were only included, as they were the studies that reported empirical evidence of relations between climate change-related exposures and pediatric health outcomes. The eligibility criteria for studies included those that provided quantitative data indicating a relationship between exposures such as temperature change, air pollutants, humidity, or extreme weather conditions and health outcomes among children aged 0 to 18 years. In addition, only studies that provided quantifiable effect measures, such as relative risks, odds ratios, or incidence rate ratios, along with associated confidence intervals, were deemed suitable for the analysis.

Any publication had to be in English, with full texts accessible, to extract and appraise information properly. Articles that were limited to adult samples, review articles, or editorials without primary research, and articles that did not provide quantitative findings or sufficiently covered statistics were eliminated. These criteria ensured that the dataset was empirically sound and consistent with the study’s analytical needs.

### Data extraction and data management

Data extraction was systematic, using a standardized form developed in Microsoft Excel. All the studies included had their corresponding details, including author details, year of publication, country of study, study design, population characteristics, and exposure type. The information on exposure measures, time, and methods of measurement was recorded to enable comparisons across various climatic and environmental conditions. Pediatric health outcome information was also gathered, including respiratory diseases, heat-related illnesses, infectious diseases, and other measures of morbidity, along with statistical measures of association and their 95% confidence intervals.

Methodological consistency to minimize confounding bias has been adopted by obtaining effect estimates from the most adjusted models available in each study. Where appropriate, relative risks or odds ratios were log-transformed, and standard errors were estimated to harmonize the data across studies. Data extraction was performed by two independent reviewers, and the entries were cross-verified to achieve a high degree of accuracy and completeness. Any inconsistencies were addressed through collective consultation and agreement.

### Quality and risk-of-bias assessment

Quality assessment was performed to determine the validity of the studies included to determine the internal validity and methodological rigor of the study through the use of the Newcastle Ottawa Scale (NOS), as this tool is extensively used in observational studies. The NOS evaluates studies in three areas, including participant selection, comparability of study groups, and the determination of exposure and outcomes. The score was allocated to each study according to the following criteria, the higher the score, the better the methodological quality and the less risk of bias. The studies with a score of 7 and more were taken to be of high quality.

Besides that, the Risk of Bias in Non-Randomized Studies of Interventions (ROBINS-I) instrument was used to give a domain-based analysis of possible biases, such as confounding, selection bias, exposure classification, missing data, outcome measurement, and selective reporting. Instead of producing a numerical score, ROBINS-I has been able to have qualitative classification of studies as either low, moderate, or serious risk-of-bias.

These evaluations were to provide a contextual understanding of the results of findings during synthesis. The lower quality of the studies and the risk of bias were considered during the interpretation that implied the caution of the study and methodological limitations during the conclusion. The estimates of the effects were not statistically weighted using quality assessments but rather the narrative evaluation informed the strength and consistency of the evidence base. This methodology was a way of making sure there was transparency in the approach of methodology and interpretive rigor.

### Statistical analysis

There was such methodological heterogeneity across the studies (e.g. differences in exposure definitions, short and long-term, outcome measures, respiratory morbidity, infectious diseases, heat-related illness, effect measures, age-stratification, study design, time-series, cohort, ecological, modelling) that a formal quantitative meta-analysis was not performed. A systematic narrative synthesis method was used instead to generalize and contrast results across research studies.

The effect estimates as per the results of the studies included were taken out of the most adjusted models. Relative risks and odds ratios where appropriate were analyzed qualitatively in order to determine direction, magnitude and consistency of relationship between exposure categories. The exposure type (air pollution, heat, extreme weather), geographic place, the age group and the study design were analyzed.

Python (version 3.12) along with Pandas and NumPy were used to create descriptive statistics to summarize study characteristics and quality scores as well as the regional distribution patterns. Visualization was done with Matplotlib to create heatmaps and histograms that depict the trends on methods and location. Study contextualization of risk of bias and study quality assessments was done instead of using them to weight effect estimates statistically.

This strategy allowed methodological transparency as well as maintaining the interpretability of results under the face of a high level of between-study variability.

## Results and discussions

### Study selection

**Figure 1.**
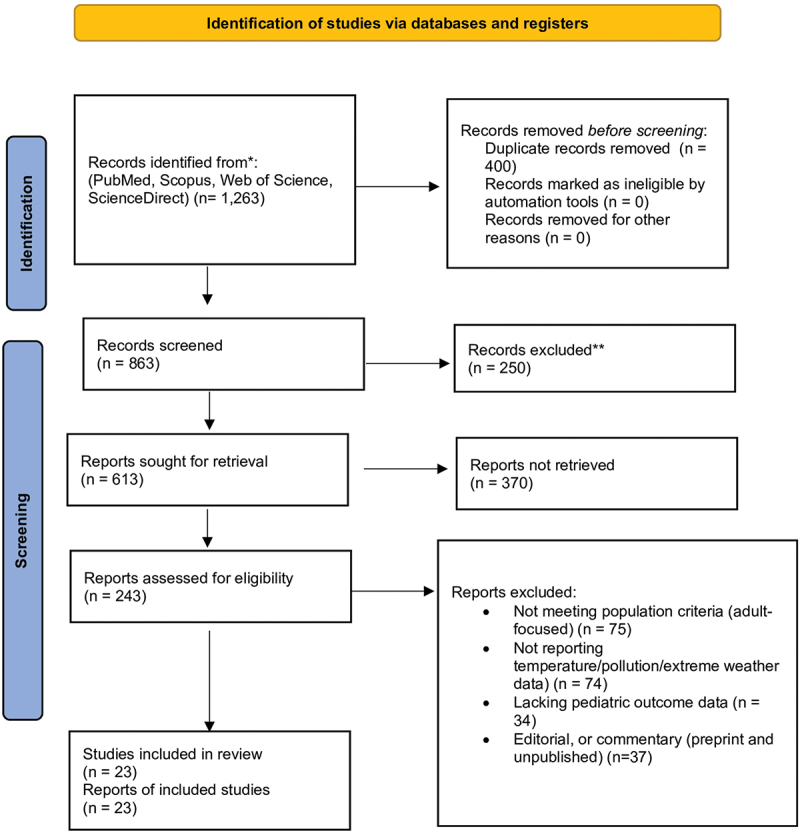
Selection of studies.

A total of 1263 records were initially identified from PubMed, Scopus, Web of Science, and ScienceDirect databases. After removing 400 duplicate records, 863 unique studies remained for screening. Figure 1 illustrates the systematic process of study identification, screening, eligibility assessment, and inclusion in accordance with the PRISMA 2020 guidelines. During the title and abstract screening stage, 250 records were excluded for failing to meet the inclusion criteria. The remaining 613 studies were sought for full-text retrieval; however, 370 could not be retrieved due to access limitations or incomplete records. Subsequently, 243 full-text articles were assessed for eligibility. Of these, 220 were excluded for several reasons, including adult-focused populations (*n* = 75), absence of relevant environmental exposure data such as temperature, air pollution, or extreme weather (*n* = 74), lack of pediatric-specific outcome data (*n* = 34), non-published, preprints research types such as commentaries, or editorials (*n* = 37). Ultimately, 23 studies were used. This rigorous selection process ensured that only methodologically sound, thematically relevant studies examining the impact of climate change on pediatric health outcomes were included in the review.

### Study characteristics

The synthesis of the 23 studies demonstrates overwhelming evidence of the effects of climate change on pediatric health, expressed through multiple complex environmental pathways. The strongest and most frequent relationship is the one between air pollution and respiratory health outcomes in children. Research like that by Zanobetti et al. [17], Gauderman et al. [18], and Ni R et al. [19] give solid proof that short-term and long-term exposure to fine particulate matter (PM2.5), nitrogen dioxide (NO2), and ozone (O3) are significant contributors to childhood asthma development and exacerbation, reduced lung function, and a high number of emergency department visits in the respiratory diseases field. The longitudinal and multicity studies show a negative correlation between early-life pollutant exposure and the subsequent development of long-lasting respiratory diseases, whereas better air quality in the future significantly correlates with quantifiable improvements in children’s pulmonary development. The presence of a significant disease burden from exposure to ambient PM2.5 is further estimated in global modeling studies, demonstrating the global scope of the issue. Table 1 explains the characteristics of the chosen studies.

**Table 1.** Characteristics of studies.

No	Author	Design	Population (age/size)	Exposure(s)	Outcome(s)	Key findings
1	Zanobetti et al. 2024	Prospective multicohort/pooled cohort analysis	Children followed from birth; *N* ≈ 5,279 across cohorts; ages: early childhood to middle childhood.	Early-life PM_2.5_, NO_2_ (mean over first 3 years)	Incident physician-diagnosed asthma (early & middle childhood)	Early-life NO_2_ and PM_2.5_ associated with increased asthma incidence; higher risks in socioeconomically disadvantaged/minoritized groups.
2	Gauderman et al. 2015	Longitudinal cohort (repeated cross-sectional cohorts)	Schoolchildren followed across cohorts (several thousand across cohorts; ages ~11–15 across follow-ups)	Long-term ambient NO_2_, PM_2.5_, PM_10_; temporal declines in pollutants	Lung function growth (FEV_1_, FVC)	Long-term improvements in air quality associated with statistically and clinically significant improvements in lung-function growth.
3	Uibel et al. 2022	Scoping review/synthesis of pediatric heat studies	Pediatric populations across included studies (infants and adolescents)	Extreme ambient temperature/heatwaves	Multiple morbidity outcomes – heat-related illness, dehydration, infectious disease, respiratory events, injuries	High/extreme heat associated with increased pediatric heat-related illness, dehydration/electrolyte imbalance, some respiratory & infectious outcomes; mixed evidence for renal/cardiac outcomes.
4	Wang et al. 2023	Cross-sectional analysis using Demographic & Health Surveys (DHS) + Dartmouth Flood Observatory	Children < 5 years; *N* ≈ 639,250 (clusters across surveys 2009–2019)	Flood exposure (event timing, duration; floods preceded by drought)	Diarrhea prevalence (caregiver-reported)	Flood exposure associated with higher diarrhea prevalence; stronger for long floods, extreme floods, and floods after drought.
5	He et al. 2022	Time-series/multicity analysis	Pediatric emergency department visits dataset (large multisite sample)	Short-term spikes in PM_2.5_, O_3_, NO_2_, etc.	ED visits for respiratory conditions (and other cardiorespiratory outcomes)	Short-term pollutant increases associated with higher pediatric ED visits, particularly for respiratory outcomes. (Large multi-site evidence)
6	Strosnider et al. 2019	Time-series, multi-county ecological study	ED visit data stratified by age (including children)	Ozone (O_3_), PM_2.5_	Respiratory ED visits (age-stratified)	Demonstrates age-specific increases in respiratory ED visits with ozone and PM_2.5_, with children showing distinct risk patterns.
7	Li et al. 2020	Prospective cohort	Schoolchildren (age range reported in paper – approx. school-age children; cohort N reported in full text)	Long-term PM_2.5_	Lung function measures (FEV_1_, FVC, FEF)	Chronic PM_2.5_ exposure associated with lower lung function in schoolchildren and altered lung-function growth.
8	Zhu et al. 2017	Time-series/case-crossover style analysis	Children (typically 0–14 or 0–18 depending on hospital records)	Short-term PM_2.5_, PM_10_, NO_2_, SO_2_, O_3_	Lower respiratory diseases (hospital admissions/ED visits)	Short-term elevations in pollutants associated with increased lower respiratory disease events in children.
9	Szyszkowicz et al. 2018	Time-series/ecological	Pediatric ED visit strata included	PM_2.5_, O_3_, NO_2_ (short-term)	Upper & lower respiratory ED visits	Short-term pollution peaks linked to rises in pediatric respiratory ED visits (upper & lower).
10	Krall al. 2018	Multicity time-series	All-ages with pediatric subgroup analysis	PM_2.5_, O_3_, NO_2_, etc.	Cardiorespiratory ED visits (including pediatric respiratory)	Multicity evidence of pollutant-related increases in cardiorespiratory visits; children show elevated respiratory sensitivity.
11	Acosta-España et al. 2024	Review/systematic review	Children included within population assessment	Flood events (water contamination, displacement)	Infectious disease outbreaks (gastroenteric, vector-borne, waterborne)	Floods increase risk of infectious disease outbreaks; children often disproportionately affected due to exposure and immunity.
12	Ni et al. 2025	Global burden/ecological/modelling study	Global pediatric population (age groups as defined in paper)	PM_2.5_ exposure (ambient)	Pediatric respiratory infection burden (cases, DALYs)	Estimates a substantial attributable burden of pediatric respiratory infections to ambient PM_2.5_ worldwide.
13	Teyton et al. 2023	Time-series/cohort of infant ED visits	Infants (age 0–1 year) sample detailed in full text	PM_2.5_ (and other pollutants)	ED visits in infancy (respiratory, other causes)	PM_2.5_ exposure associated with increased risk of ED visits during first year of life.
14	Schapiro et al. 2024	Narrative/thematic review	Children across life stages	Heat, air pollution, extreme weather, infectious disease pathways	Review of child-specific pathways linking climate change to health outcomes; identifies vulnerabilities & policy needs.	(Seminal review; PMC available)
15	Pacheco et al. 2020	Thematic review/commentary	Children (various age groups)	Multiple climate drivers (heat, drought, air quality, extreme weather)	Multiple health outcomes (nutrition, infections, developmental impacts)	Synthesizes evidence for broad, multi-sectoral child health impacts from climate change.
16	Dimitrova et al. 2022	Observational/modelling/epidemiologic analyses	Pediatric populations within affected regions (age groups per paper)	Precipitation variability (floods, drought)	Infectious disease incidence in children (enteric, vector-borne)	Precipitation variability linked to changes in infectious disease risk; contexts and local WaSH mediate effects.
17	Aithal et al. 2023	Review	Children (vulnerable groups highlighted)	Ambient air pollution (PM_2.5_, O_3_, NO_2_, wildfire smoke)	Respiratory diseases, exacerbations, developmental effects	Summarizes pediatric vulnerabilities and recent evidence linking air quality to childhood respiratory outcomes.
18	Weeda et al. 2024	Systematic review/quantitative synthesis	Children (varied ages)	Climate change drivers (heat, air pollution, extreme events)	Outcomes: preterm birth, heat illness, infections, respiratory morbidity	Quantifies effect sizes where possible; highlights gaps and magnitude of child health impacts.
19	Gutiérrez et al. 2024	Time-series/health services analysis	Pediatric urgent care/ED attendances (age groups reported in paper)	Ambient temperature/heat waves	Febrile/urgent consultations, febrile illness visits	Heat associated with increases in pediatric urgent care visits for febrile illnesses.
20	Ni et al. 2024	Pooled analyses/cohort meta-analysis	All ages with pediatric subgroup analyses	Long-term PM_2.5_ exposure	Asthma incidence/prevalence (age-stratified)	Long-term PM_2.5_ exposure associated with increased asthma risk across ages, including children.
21	Hao et al. 2023	Large prospective cohort	Population-level cohort (national registry; pediatric inference possible in subgroups)	Long-term PM_2.5_	Mortality & various health outcomes; pediatric implications discussed	Large national cohort showing associations of long-term PM_2.5_ with adverse health outcomes; informs pediatric risk inference.
22	Li et al. 2015	Review article	Heat waves/ambient temperature extremes	Morbidity (heat illnesses, hospitalizations, ED visits), including pediatric outcomes	Summarizes evidence and research gaps on heat-related morbidity and children’s vulnerability.	(MDPI review)
23	Ding et al. 2019	Time-trend ecological/event-based analysis	General population with child-specific data when available	Flood events	Infectious disease incidence spikes (enteric, vector-borne)	Flood events linked to increases in certain infectious diseases that affect children (timing/susceptibility highlighted).

The second theme that dominates is the effect of extreme heat and temperature variation on children’s health. Documented literature on heatwaves and elevated ambient temperatures, including Uibel et al. [20], Gutierrez et al. [21], and Li M et al. [22], also proves that high ambient temperature and heatwaves pose a risk of heat-related diseases, dehydration, and febrile accidents among children. Infants and young children are highly vulnerable because their thermoregulatory systems are immature and they produce more heat relative to their body size. Thematic and narrative reviews indicate that physiological vulnerability is enhanced by socioeconomic and environmental factors, including poor housing, limited access to cooling facilities, and urban heat island effects. These results suggest that pediatric morbidity related to temperature will increase with further climate change, especially in low-resource, densely populated areas.

The third alarming trend observed in the evidence is associated with extreme weather events, particularly floods and precipitation variations, which are closely linked to higher rates of infectious disease outbreaks in children. Extensive studies by Wang et al. [23] and Ding et al. [24] show that children exposed to floods have a much higher rate of diarrheal disease, particularly when floods are long and follow droughts. Additional evidence for the role of floods in disseminating waterborne, vector-borne, and enteric infections is provided by the complementary data of Acosta-Espana et al. [25] and Dimitrova et al. [26], who indicate that water contamination and displacement are only some of the mechanisms driving their spread. Such studies repeatedly suggest that the scale of health effects is mediated by local infrastructure, especially water, sanitation, and hygiene (WaSH) systems, meaning that the rate of flood-related illnesses is disproportionately higher in areas of low vulnerability and resources.

Another comprehensive, integrative theme across some review studies, such as those by Pacheco et al. [27], Aithal et al. [28], and Wright et al. [29], is the multi-pathway effects of climate change on child health. These reviews provide evidence synthesis on the relationships between heat and air pollution and extreme weather, and their diverse health impacts, including respiratory diseases, infections, nutritional deficiencies, and developmental consequences. They all underline the idea that the effects of climate change are cumulative and synergistic, but not isolated, as they often interact at the biological, environmental, and social levels. The fact that some reviews included quantitative estimates of the effects of climate change also supports the severity of the risks to children’s well-being.

### Biological mechanisms underlying pediatric vulnerability

The biological factors that exacerbate the susceptibility of children to the effects of environmental exposures to climate are the physiological, developmental, and immunological factors. In the case of air pollutants, e.g. PM2.5 and NO2, the children breathe more air per kilogram of body weight than adults do, which means that they absorb larger proportions of the pollutants. The lungs and immune system of them are still underdeveloped and are more susceptible to oxidative stress, airway inflammation, and structural airway remodeling.

Traffic-related air pollution (TRAP) and especially NO2 and fine particulates have been linked with greater amounts of reactive oxygen species (ROS) production, inflammatory pathway activation, and dysfunction of epithelial barriers in the airways. These are the mechanisms that lead to airway hyperresponsiveness and higher levels of asthma. New epidemiological data also reveal that prenatal exposure to TRAP is going to be linked with an increased risk of childhood asthma, respiratory infections and even a greater vulnerability to viral respiratory diseases. It is believed that oxidative stress-mediated pathways and immune dysregulation are the key biological processes that connect the exposure to pollutants and chronic childhood respiratory morbidity.

Besides direct respiratory toxicity, air pollution could also affect long-term immune programming at critical stages of development, which can make it more prone to allergic diseases. These mechanistic observations support the uniformity of the epidemiological evidence that is synthesized in this review.

### Indirect pathways: the role of the indoor and human microbiome

In addition to direct toxicological effects, there has been an emerging evidence that shows environmental pollutants could affect the health of pediatrics indirectly by modifying the interior and human microbiome. PM2.5 and NO2 pollution of the air may alter the diversity and composition of indoor microbes through its impact on the dynamics of ventilation, deposition of particles, humidity, and growth of the microbes. Fu et al. [30] also emphasized theoretical achievements that connect the change of indoor microbiota to environmental change to the origin of allergies and immune-mediated diseases, which underline the importance of microbial dysbiosis in the early immune formation. Added to this, Sun et al. [31] presented empirical data to show that indoor microorganisms may mediate the relationship between air pollution exposure and rhinitis in preschool children and imply a completely mediated pathway in some settings.

These results suggest that environmental changes caused by the impact of climate change can affect the health of children not just due to direct inflammatory stress-inducing and oxidative stress but also by causing ecological disturbances in the built environments [32]. Given that early childhood is a pivotal time to program the immune to respond to different microorganisms, pathways through microbiome can enhance susceptibility to asthma, allergic rhinitis, and other immune-related disorders [33]. The application of microbiome lenses to climate-health studies provides a mechanistic framework for greater insight on childhood vulnerability.

### Quality assessment

The Newcastle-Ottawa Scale (NOS) and ROBINS-I tool were applied to assess the quality of the methodology of the 23 articles included in the study and to determine internal validity and risk of bias. These instruments were applied to give information about the interpretive power of the evidence in the narrative synthesis as opposed to quantitative estimates.

**Figure 2.**
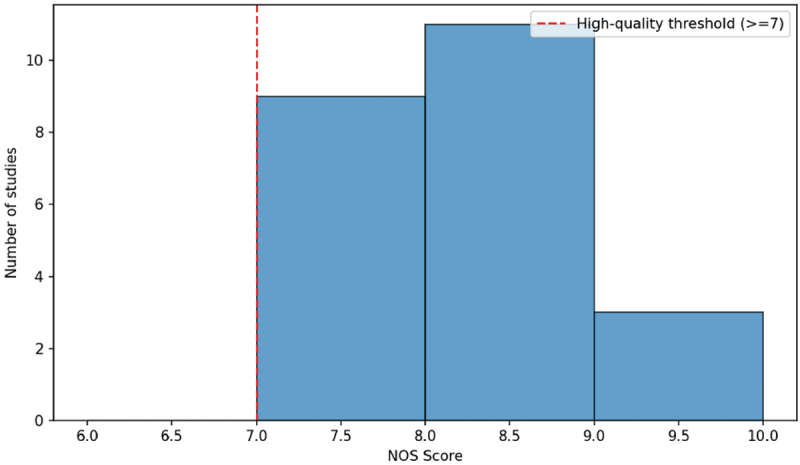
Distribution of NOS score.

Most studies were found to have a NOS score of between 7 and 9 as depicted in Figure 2 thus the evidence base had an overall high methodological quality. The fact that the scores are concentrated in this range is an indication that the sample selection procedures, intergroup comparability and the outcome assessment are adequate in most studies. The other small group of studies scored lower mainly because of the limitation of confounding control or selection of participants.

**Figure 3.**
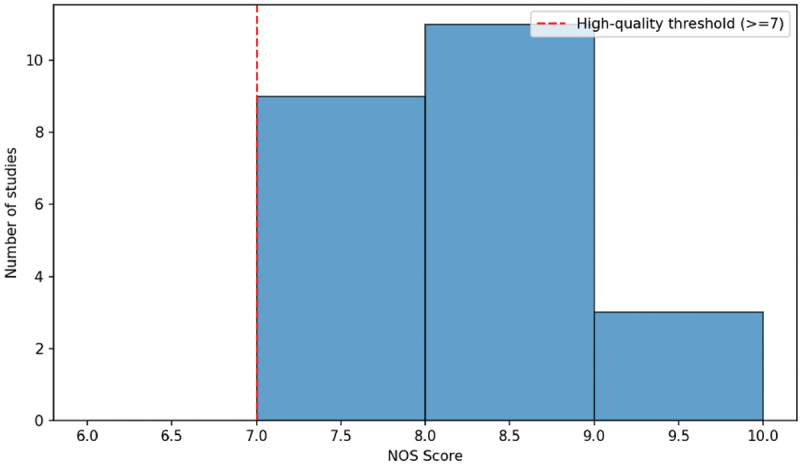
ROBINS-I risk of bias.

The ROBINS-I assessment (Figure 3) also suggested that about half of the studies were rated as low risk of bias (*n* = 12), and the rest of the studies as moderate risk of bias (*n* = 11). There were no studies that were rated as having critical risk of bias. A moderate risk rating was commonly linked to the use of retrospective data, or the incompleteness of adjustment to the possible confounding factors, including such factors as socioeconomic status and co-exposure to other environmental pollutants [34].

The findings of the NOS and the ROBINS-I tests give reason to believe that the narrative synthesis provided in the following sections is founded on the studies with high design strength and that internal validity is acceptable.

### Geographical and exposure patterns

**Figure 4.**
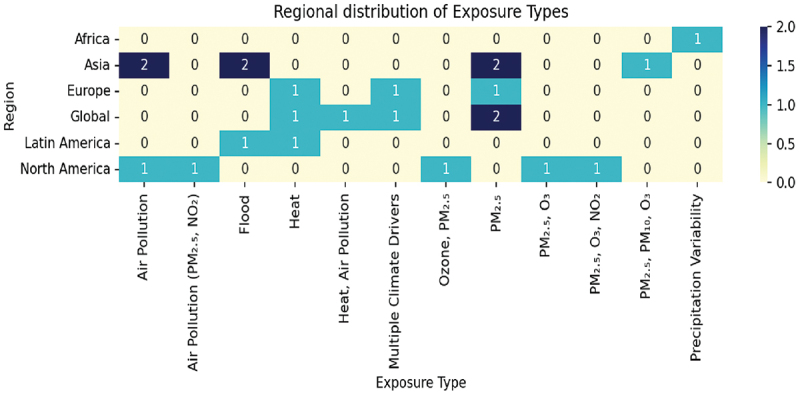
Regional distribution of exposure types.

Geographical distribution of studies is such that the studies that have been done are more concentrated in North America, Europe and Asia with relatively low representation among the African and South Americans. Figure 4 gives an overall description of types of exposure investigated by region. Exposures related to temperature were more commonly researched in North America and Europe, and air pollution exposures especially of particulate matter were more common in studies carried out in Asia [35]. Such distribution is likely to be indicative of both climatic specifics of the region and variations in infrastructural research and monitoring priorities [36].

Despite the fact that most primary studies considered individual exposures (e.g. air pollution, heat or floods), in real-world climate it is common to have interacting and compound exposures [37]. An illustration of this is that higher temperatures may enhance ozone formation, wildfire-related particulate matter may be exacerbated by drought, and heat stress and displacement may be coupled with flooding [38]. These co-exposures can effectuate synergies between biological and social processes to increase the pediatric risk to their health. The fact that most of the literature utilizes single-exposure models indicates that cumulative risk associated with climate can be underestimated [39]. The concern of future research efforts should focus on multi-exposure modeling models in order to capture such complicated interactions.

### Methodological patterns

**Figure 5.**
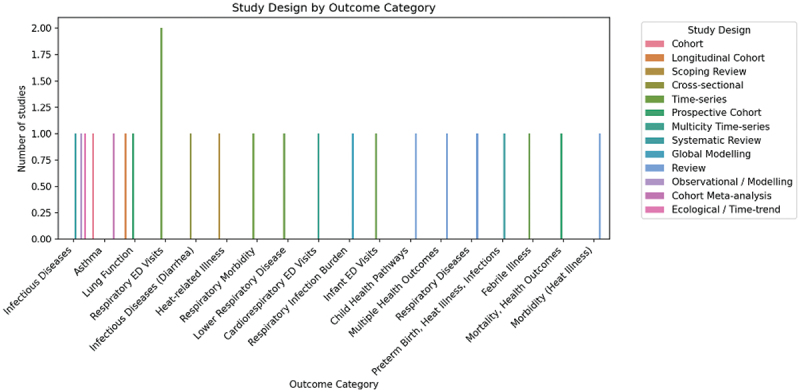
Study design by outcome category.

The description of the distribution of study designs based on the outcome category is summarized in Figure 5. The most common designs were time-series and case-crossover designs, which were applicable to short-term outcomes, such as emergency department visits, respiratory exacerbation, and heat-related morbidity. Conversely, cohort and cross-sectional designs were more extensively utilized in the study of chronic or developmental effects of the long-term environmental exposures.

Such methodological distinction is explained by the necessity to match study design with exposure and latency of outcome. The variety of designs also added to the heterogeneity between studies, which justified the choice of a structured narrative synthesis.

### Regional quality comparison

**Figure 6.**
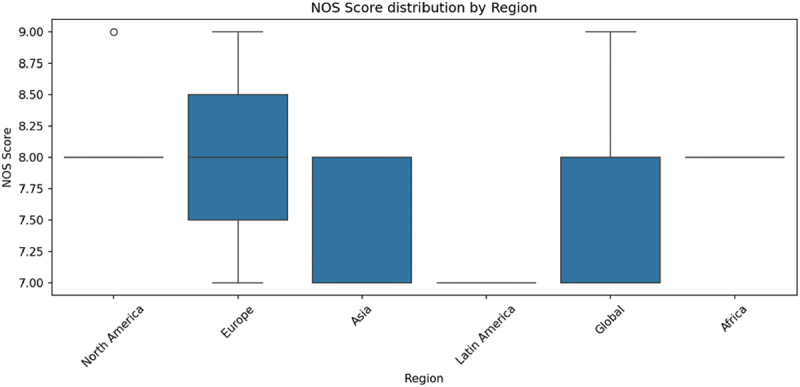
NOS score distribution by region.

Figure 6 makes comparison of NOS scores by geographic regions. Median quality scores and comparatively low variability were established in the studies carried out in Europe and North America and were probably due to more standard research protocols and more available data on environmental monitoring. Conversely, the interquartile ranges were wider in Asian and developing parts of the studies and imply that there were more heterogeneities in the study designs and data.

These geographic inequalities underscore the importance of harmonizing methodologies and better environmental surveillance systems and capacity-building research in underrepresented regions to increase global comparability of evidence.

### Policy implications

Adaptive public health methods should be able to convert epidemiological evidence into specific interventions. Some of these successful strategies are school-level heat action plans adjusting outdoor activity schedules in response to extreme heat events, city greening, and enhanced air quality monitoring systems combined with early warning systems about vulnerable pediatric populations. Resilience of water, sanitation and hygiene (WaSH) has also proven to be effective in curbing the outbreak of diarrhea after floods among children. There is a specific shortage in capacity-building and environmental surveillance systems in underrepresented areas like Africa and South America to curb the research and intervention gaps identified.

## Conclusion

The research provides strong evidence that climate change significantly affects children’s health through mechanisms such as air pollution, excessive heat, and floods. The final synthesis of 23 high-quality studies indicates reproducible correlations between climatic exposures and the risks of respiratory diseases, heat-related diseases, and infections among children. These findings are credible because the methodological rigor, as indicated by high NOS and ROBINS-I scores, is strong. Nevertheless, the issue of regional gaps in research representation underscores the urgent need to use data from low- and middle-income countries. The findings underscore the fact that climate change has a disproportionate impact on socioeconomically disadvantaged children, which contributes to the inequity in health globally. Environmental policy, healthcare adaptation, and community resilience are integrated strategies to protect pediatric populations. In conclusion, this study highlights the importance of protecting children’s health in the warming world as a scientific need, as well as a moral and social imperative that requires an urgent international response.

## Supplementary Material

Checklist.docx

## Data Availability

All data used in this review were obtained from previously published studies. The compiled dataset generated from these studies is available upon reasonable request from the corresponding author.
